# Clinical and molecular characterization of a multi-institutional cohort of pediatric spinal cord low-grade gliomas

**DOI:** 10.1093/noajnl/vdaa103

**Published:** 2020-08-24

**Authors:** Sydney T Grob, Liana Nobre, Kristen R Campbell, Kurtis D Davies, Scott Ryall, Dara L Aisner, Lindsey Hoffman, Shadi Zahedi, Andrew Morin, Michele Crespo, Anandani Nellan, Adam L Green, Nicholas Foreman, Rajeev Vibhakar, Todd C Hankinson, Michael H Handler, Cynthia Hawkins, Uri Tabori, B K Kleinschmidt-DeMasters, Jean M Mulcahy Levy

**Affiliations:** 1 Department of Pediatrics, University of Colorado Denver, Aurora, Colorado, USA; 2 The Morgan Adams Foundation Pediatric Brain Tumor Research Program, Children’s Hospital Colorado, Aurora, Colorado, USA; 3 Department of Hematology and Oncology, Hospital for Sick Children, Toronto, Ontario, Canada; 4 Department of Pathology, University of Colorado Denver, Aurora, Colorado, USA; 5 The Arthur and Sonia Labatt Brain Tumour Research Centre, The Hospital for Sick Children, Toronto, Ontario, Canada; 6 Center for Cancer and Blood Disorders, Phoenix Children’s Hospital, Phoenix, Arizona, USA; 7 Department of Neurosurgery, University of Colorado Denver, Aurora, Colorado, USA; 8 Department of Laboratory Medicine and Pathobiology, University of Toronto, Toronto, Ontario, Canada; 9 Department of Pediatric Laboratory Medicine, The Hospital for Sick Children, Toronto, Ontario, Canada

**Keywords:** BRAF, low-grade glioma, spine

## Abstract

**Background:**

The mitogen-activated protein kinases/extracelluar signal-regulated kinases pathway is involved in cell growth and proliferation, and mutations in *BRAF* have made it an oncogene of interest in pediatric cancer. Previous studies found that *BRAF* mutations as well as *KIAA1549–BRAF* fusions are common in intracranial low-grade gliomas (LGGs). Fewer studies have tested for the presence of these genetic changes in spinal LGGs. The aim of this study was to better understand the prevalence of BRAF and other genetic aberrations in spinal LGG.

**Methods:**

We retrospectively analyzed 46 spinal gliomas from patients aged 1–25 years from Children’s Hospital Colorado (CHCO) and The Hospital for Sick Children (SickKids). CHCO utilized a 67-gene panel that assessed *BRAF* and additionally screened for other possible genetic abnormalities of interest. At SickKids, *BRAF*^*V600E*^ was assessed by droplet digital polymerase chain reaction and immunohistochemistry. BRAF fusions were detected by fluorescence in situ hybridization, reverse transcription polymerase chain reaction, or NanoString platform. Data were correlated with clinical information.

**Results:**

Of 31 samples with complete fusion analysis, 13 (42%) harbored *KIAA1549–BRAF*. All 13 (100%) patients with confirmed *KIAA1549–BRAF* survived the entirety of the study period (median [interquartile range] follow-up time: 47 months [27–85 months]) and 15 (83.3%) fusion-negative patients survived (follow-up time: 37.5 months [19.8–69.5 months]). Other mutations of interest were also identified in this patient cohort including *BRAF*^*V600E*^, *PTPN11*, *H3F3A*, *TP53*, *FGFR1*, and *CDKN2A* deletion.

**Conclusion:**

*KIAA1549–BRAF* was seen in higher frequency than *BRAF*^*V600E*^ or other genetic aberrations in pediatric spinal LGGs and experienced lower death rates compared to *KIAA1549–BRAF* negative patients, although this was not statistically significant.

**Key Points**

- *KIAA1549–BRAF* fusion was the most common aberration identified in the low-grade glioma cohort.
- Patients with *KIAA1549–BRAF* fusion had excellent long-term survival.
- Spinal cord low-grade tumors may be good candidates for MEK inhibition.

**Importance of the Study**

Intracranial low-grade glioma (LGG) in the pediatric population has been well researched for the genetic aberrations that define this population. They are known to have *BRAF* mutations as well as *KIAA1549–BRAF* fusions, which is helpful in predicting outcome and treatment responses in this group. Fewer studies have evaluated spinal cord LGG for similar genetic aberrations. This study retrospectively investigated the genetic landscape of a cohort of pediatric spinal LGGs for the presence of *BRAF* aberrations as well as other genetic mutations of interest. *BRAF* fusions were the most prevalent change identified. All patients with this fusion had excellent survival. This pediatric population suffers from severe complications as a result of treatment needed to maintain tumor control, and better understanding the genetic landscape of this tumor could potentially inform treatment and predict long-term outcomes in this population.

Low-grade gliomas (LGGs) of the spinal cord are rare in pediatric patients. Tumors arising in the central nervous system (CNS) make up 20% of all pediatric cancer, but intramedullary spinal cord tumors are only 2%–10% of CNS lesions.^1^ Patients diagnosed with spinal cord LGG have a high overall survival (OS) with progression-free survival (PFS) ranging from 34% to 88%.^2^ Despite low mortality, there is high morbidity associated with spinal LGG treatment. Specifically, patients can suffer from significant neurologic and orthopedic complications from surgery, chemotherapy, and radiation. Defining the mutational characteristics of spinal cord LGG could provide predictive information regarding clinical outcomes and potential therapy options.

The most common genetic changes in intracranial LGGs or glioneuronal tumors involve the mitogen-activated protein kinases/extracelluar signal-regulated kinases (MAPK/ERK) pathway,^3–6^ which drives a number of processes including cellular proliferation, differentiation, mortality, stress response, apoptosis, and survival.^7^ There are a number of mutations and fusions in this pathway that result in constitutive over-activation of the MAP/ERK pathway.^8^

Cerebellar pilocytic astrocytomas often exhibit MAPK/ERK hyperactivation as a consequence of a *KIAA1549–BRAF* fusion.^3,4^ LGGs occurring elsewhere in the brain have a higher percentage of tumors with a point mutation in *BRAF* at codon 600 (*BRAF*^*V600E*^ and variants such as *BRAF*^*V600D*^). This mutation is also seen in many CNS tumors including desmoplastic infantile gangliogliomas, diffuse astrocytomas, gangliogliomas, pleomorphic xanthoastrocytomas, and epitheloid glioblastomas.^9^

At present, gross total surgical resection (GTR) is the first line of treatment for LGG, but adjuvant therapy could include chemotherapy (ie, carboplatin, vincristine, and vinblastine) and potentially radiation therapy. These are particularly considered if GTR is not feasible.^10^  *BRAF*^*V600E*^ inhibitors like vemurafenib (NCT01748149) and dabrafenib (NCT01677741) and MEK inhibitors like trametinib (NCT03434262) are being explored to treat these tumors in phase 1 and 2 trials. Tumors with the *KIAA1549–BRAF* fusion are considered to be RAS-independent and are resistant to first-generation RAF inhibitors (vemurafenib and dabrafenib).^11,12^ This makes MEK inhibition a better option for these patients. In addition to the potential for the development of resistance to targeted therapies,^5,11,13,14^ progression following single- and multi-agent chemotherapy remains a concern in these patients with 5-year PFS estimated between 43% and 53.2%.^15–17^

To identify potential new therapy options in pediatric low-grade spinal cord tumors, we evaluated whether these tumors identified at 2 institutions (Children’s Hospital Colorado [CHCO] and SickKids) harbor targetable lesions such as the *KIAA1549–BRAF* fusion and *BRAF*^*V600E*^ mutations. Sixty-seven secondary genes were also screened for mutations for the tumors from CHCO. We evaluated clinical data and performed genetic testing on 46 spinal LGG (WHO grade 1 or 2) with available tumor samples and mapped PFS and OS of these patients to see if an association between aberrations in *BRAF*, treatment, and OS could be identified.

## Materials and Methods

### Patients and Inclusion Criteria

Fifty-five patients were identified as having intramedullary spinal cord tumors between 1995 and 2016. Of these patients, 46 patients ranging in age from 1 to 25 years (median age at diagnosis: 9.5) had a confirmed WHO grade 1 or 2 spinal cord tumor at CHCO (Aurora, CO) and SickKids (Toronto, ON). Nine patients from CHCO were excluded from analysis after it was determined that they had high-grade spinal cord malignancies. Of note, samples included from SickKids were recently also analyzed in a wider study of LGG in multiple CNS locations.^18^

Patient charts were retrospectively analyzed for age, diagnosis, location of tumor, past genetic tests completed on a patient’s tumor, treatment, subsequent relapses, treatment at relapse, and date of last follow-up or death. Pathology reports and imaging for each patient were subsequently analyzed to verify an accurate diagnosis, location of tumor, and genetics run at the time of biopsy/resection at CHCO. SickKids considered initial diagnosis and subsequent treatments for each patient.

### Institutional Review Board Approval of Patient Specimens

Primary patient samples were obtained and collected from CHCO and SickKids in accordance with local and federal human research protection guidelines and institutional review board (IRB) regulations. Ethical standards according to the Helsinki Declaration were followed and the work was approved by local IRB committees (CHCO approvals: COMIRB 95-500 and COMIRB 05-0149; SickKids approval: REB1000030563). Written informed consent was obtained for all specimens collected.

### Statistical Analysis

Baseline characteristics were reported as count and proportion (%) or median (interquartile range [IQR]), separately by grade 1 and 2 tumors. Time to death was defined as the time from diagnosis to death from any cause or time from diagnosis to the last follow-up if a patient survived. Time to progression was defined as the time from diagnosis to tumor progression or death, with censoring also at the last follow-up date. OS and PFS were estimated at time points of interest using Kaplan–Meier curves. Cox proportional hazard models were fit for time to death and time to progression, and log-rank statistics were reported for differences between tumor grade and KIAA1549–BRAF positivity. All statistical analysis was performed in R version 3.6.1.

### Mutational and Gene Fusion Analysis

Samples at CHCO were analyzed as follows. Total nucleic acid (TNA) was extracted in a CLIA-certified laboratory from formalin-fixed, paraffin-embedded (FFPE) processed material (*n* = 15) or from frozen material (*n* = 7) using the Agencourt FormaPure Kit (Beckman Coulter). For mutational analysis, TNA was then processed via the Archer VariantPlex Solid Tumor library preparation kit that is designed to amplify selected regions in 67 genes (ArcherDx). All manufacturer-recommended cutoffs for quality control were used and only samples that met appropriate quality levels were sequenced. Libraries were sequenced via the Illumina MiSeq or Illumina NextSeq (Illumina). Raw sequence data were processed for mutational calling by using the Archer Analysis software package (version 5.1.2.2; ArcherDx). For fusion analysis, 23 FFPE and 7 frozen samples had TNA processed via the Archer FusionPlex Solid Tumor library preparation kit that is designed to detect gene fusions and novel isoforms involving selected exons of 53 genes (ArcherDx). Libraries were sequenced via the Illumina MiSeq (Illumina). Raw sequence data were processed for fusion calling by using the Archer Analysis software package (version 4.1.1.7; ArcherDx). SickKids analyzed FFPE samples (*n* = 18) as previously described.^18^ In short, *KIAA1549–BRAF* fusion was tested by NanoString, reverse transcription polymerase chain reaction, and fluorescence in situ hybridization. *BRAF*^*V600E*^ was tested by droplet digital polymerase chain reaction (ddPCR) and immunohistochemistry. Other genetic alterations were tested by Sanger and ddPCR.

## Results

All patients diagnosed with an intramedullary spinal cord tumor between 1995 and 2016 at CHCO and patients with spinal LGG from SickKids were identified for potential inclusion and analysis (Table 1). Forty-six patients were identified with WHO grade 1 or 2 tumors. LGG patients were categorized into 6 different WHO grade 1 or 2 morphological diagnoses. The cohort was 59% males with a predominant diagnosis of pilocytic astrocytoma (57%). The majority of tumors were WHO grade 1 (78%). A significant number of patients had a subtotal resection (STR) or biopsy only (83%). Therapy provided included chemotherapy (50%) and radiation (17%). Additional demographics are given in Table 1.

**Table 1.** Clinical Characteristics by Tumor Grade^a^

Characteristic	All (*n* = 46)	Grade 1 (*n* = 36)	Grade 2 (*n* = 10)
Age at diagnosis, years	9.5 (4.8–12)	10 (6–12.2)	5 (4.1–11.1)
Male	27 (59%)	20 (56%)	7 (70%)
Diagnosis			
Astrocytoma	13 (28%)	6 (17%)	7 (70%)
Ganglioglioma	4 (9%)	4 (11%)	0 (0%)
Gemistocitic astrocytoma	1 (2%)	0 (0%)	1 (10%)
Glioneuronal tumor	1 (2%)	0 (0%)	1 (10%)
Low-grade glioma with pilocytic/pilomyxoid features	1 (2%)	1 (3%)	0 (0%)
Pilocytic astrocytoma	26 (57%)	25 (69%)	1 (10%)
Region of spine			
Cervical	13 (28%)	12 (33%)	1 (10%)
Cervical/thoracic	17 (37%)	12 (33%)	5 (50%)
Thoracic	11 (24%)	9 (25%)	2 (20%)
Thoracic/lumbar	5 (11%)	3 (8%)	2 (20%)
Treatment (surgery)			
Gross total resection	6 (13%)	6 (17%)	0 (0%)
Near total resection	2 (4%)	2 (6%)	0 (0%)
Subtotal resection	38 (83%)	28 (78%)	10 (100%)
Chemotherapy	23 (50%)	17 (47%)	6 (60%)
Radiation	8 (17%)	7 (19%)	1 (10%)
BRAF fusion (*N* = 31)	13 (42%)	12 (52%)	1 (12%)
Clinical outcomes			
Relapse	17 (37%)	13 (36%)	4 (40%)
Months to relapse	11 (3–24)	14 (4–26)	7 (3–14)
Death	7 (15%)	6 (17%)	1 (10%)
Months to death	26 (17–40)	23 (15–41)	34 (34–34)

^a^Numbers reported are median (interquartile range) or count (proportion).

**Figure 1.**
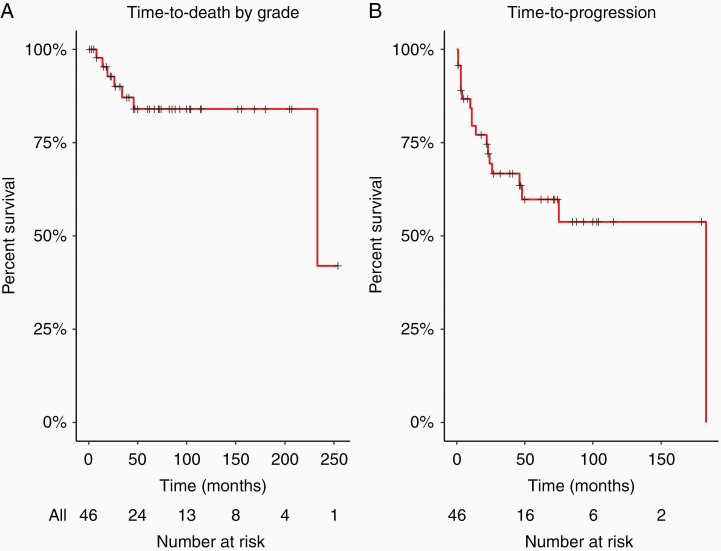
Overall survival (OS) and progression-free survival (PFS) of low-grade spinal cord tumors. (A) Time to death of grade 1/2 spinal cord tumors (5-year OS 84%), *N* = 46, censoring denoted by “+”; and (B) PFS of grade 1/2 spinal cord tumors (5-year PFS 60%), *N* = 46.

Among all participants, 85% survived the entirety of the study period (median follow-up time: 67 months, IQR [30–104]). Among the 7 (15%) who died, the median time to death was 26 months (IQR 17–40). The time-to-death and time-to-progression curves are shown in Figure 1. The estimated 5-year survival rate was 84% (95% confidence interval [CI]: 73%–97%), and the estimated 5-year PFS rate was 60% (95% CI: 46%–78%). Time to death and time to progression did not differ significantly by grade 1 versus grade 2 (*P* = .61 and *P* = .79, respectively).

**Figure 2.**
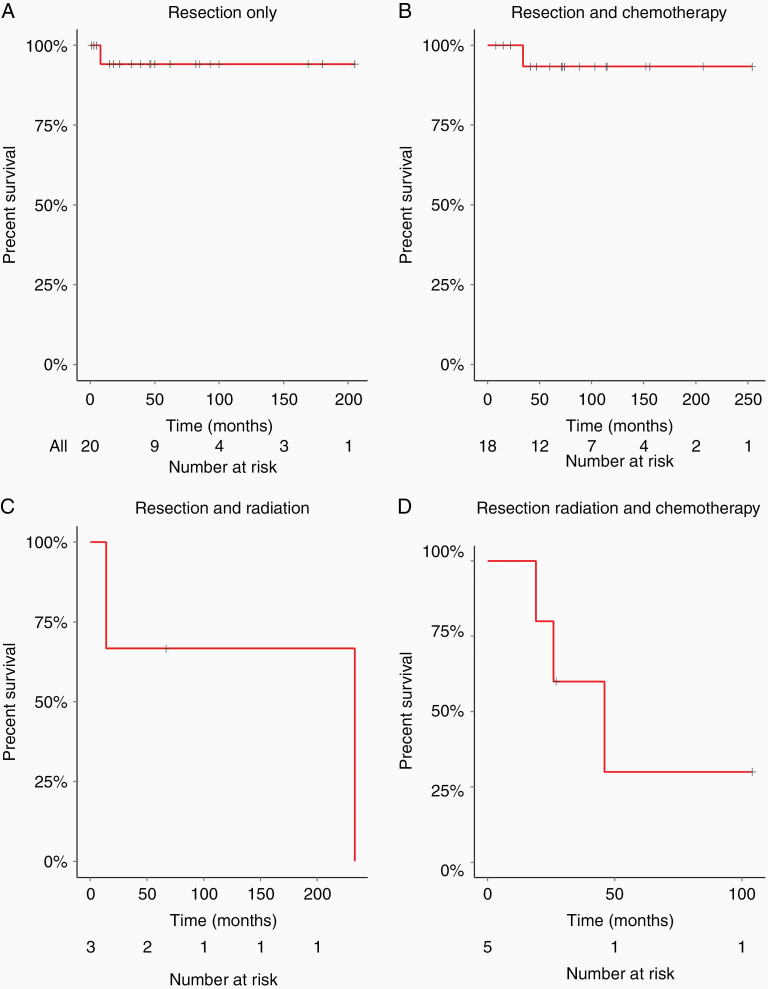
Overall survival (OS) in relation to a therapeutic treatment. (A) Time to death of grade 1/2 who had only undergone surgical resection (OS = 94%; *n* = 20). (B) Time to death of grade 1/2 who had resection and chemotherapy (OS = 93%; *n* = 18). (C) Time to death of grade 1/2 patients following resection and radiation (OS = 60%; *n* = 3). (D) Time to death of grade 1/2 patients following resection, chemotherapy, and radiation (OS = 30%; *n* = 5).

Patients treated with resection-only (Figure 2A) had the highest estimated survival rate of 94% (95% CI: 84%–100%) at 5 years. Surgical resection followed by a chemotherapy regimen resulted in an estimated 5-year survival rate of 93% (Figure 2B; 95% CI: 82%–100%). Those that underwent both surgical resection and radiation had an estimated 5-year survival rate of 67% (95% CI: 30%–100%), and those undergoing surgical resection, chemotherapy, and radiation had a 5-year survival rate of 30% (95% CI: 6%–100%).

Sixteen patients at CHCO and 18 patients from SickKids had samples with results available for secondary mutation analysis. A 67-gene panel was used to test the frozen tumor specimens at CHCO (*n* = 7; Supplementary Figure 1). Failure of analysis in some of the samples (*n* = 12) was suspected to be due to samples being very old coupled with suboptimal tissue fixation resulting in TNA that did not pass the manufacturers quality control measures. Of those analyzed, there were 9 different genetic mutations and/or fusions identified in 21 patients including mutations in *BRAF*, *PTPN11*, *H3F3A*, *TP53*, *FGFR1*, and *CDKN2A* (Table 2).

**Table 2.** Mutations Found in WHO Grade 1/2 Spinal Cord Tumors

Histology, *N* = 34	Pilocytic Astrocytoma, 20 (59%)	Gemistocitic Astrocytoma, 1 (3%)	Astrocytoma NOS, 7 (21%)	Ganglioglioma, 4 (12%)	Low-Grade Glioma Pilocytic/Pilomyxoid Features, 1 (3%)	Glioneuronal Tumor, 1 (3%)
Gene	Number (%)					
*BRAF* ^*V600E*^	0 (0%)	0 (0%)	1 (14%)	1 (25%)	0 (0%)	0 (0%)
			GOF	GOF		
*PTPN11*	0 (0%)	0 (0%)	0 (0%)	1 (25%)	0 (0%)	0 (0%)
				Asp61Tyr		
				LOF		
*H3F3A*	0 (0%)	0 (0%)	0 (0%)	1 (25%)	0 (0%)	0 (0%)
				Lys28Met		
				LOF		
				Gln6Leu		
				US		
*TP53*	0 (0%)	0 (0%)	0 (0%)	1 (25%)	0 (0%)	0 (0%)
				Arg273Cys		
				LOF		
*FGFR1*	1 (5%)	0 (0%)	1 (14%)	0 (0%)	0 (0%)	0 (0%)
	Lys656Glu		Asp546Lys			
	GOF		GOF			
*CDKN2A*	1 (10%)	0 (0%)	0 (0%)	0 (0%)	0 (0%)	0 (0%)
	LOF					
*BRAF– KIAA1549* Fusion	12 (60%)	0 (0%)	1 (14%)	0 (0%)	0 (0%)	0 (0%)
	GOF		GOF			

US, unknown significant; GOF, gain of function; LOF, loss of function.

There were thirty-one total patients in which *BRAF–KIAA1549* was successfully tested between CHCO (*N* = 13) and SickKids (*N* = 18). In total, 13 (42%) patients tested positive, 6 from CHCO and 7 from SickKids. Twelve of the patients diagnosed with the fusion had the diagnosis of pilocytic astrocytoma and one had an astrocytoma not otherwise specified (NOS; Table 2). In this patient cohort, there were 18 patients confirmed negative for the *KIAA1549–BRAF* fusion including the diagnoses: glioneuronal tumor, astrocytoma NOS, pilocytic astrocytoma, gemistocitic astrocytoma, ganglioglioma, and LGG with pilocytic/pilomyxoid features (Table 2). In agreement with previous publications, ganglioglioma is more frequently related to *BRAF*^*V600E*^ mutations.^19^ Across all tumors, there were only 2 *BRAF*^*V600E*^ mutations identified in patients diagnosed with astrocytoma NOS and ganglioglioma, respectively.

**Figure 3.**
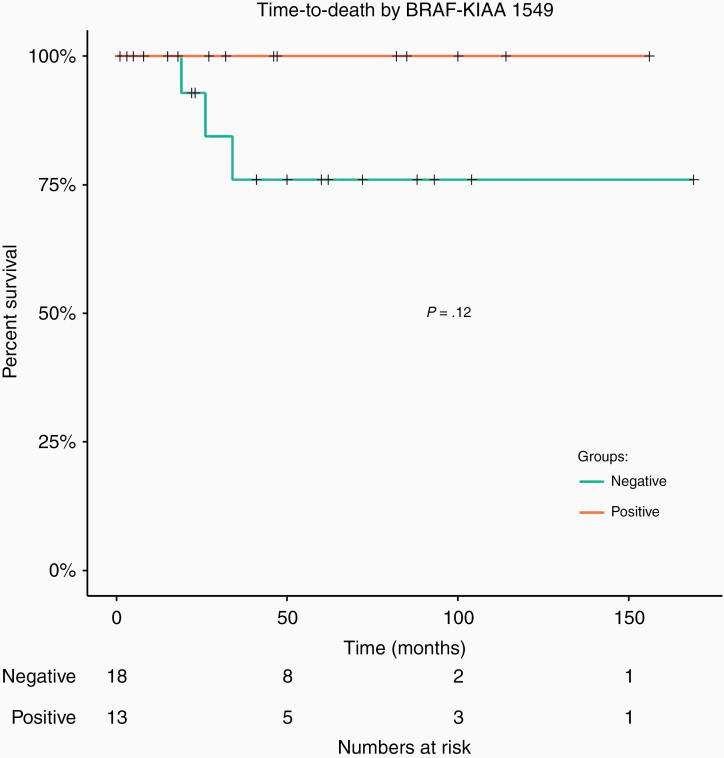
Overall survival in *KIAA1549–BRAF* fusion patients. Patients had to be tested for the *KIAA1549–BRAF* fusion (*n* = 31) in order to be included in this figure. No difference in time to death by *KIAA1549–BRAF* status (*P* = .12).

Time to death was not significantly different *KIAA1549–BRAF* fusion-positive and *KIAA1549–BRAF* fusion-negative tumors (*P* = .12; Figure 3). Interestingly, the estimated 5-year survival rate for patients who were confirmed to contain the *KIAA1549–BRAF* fusion was 100%, compared to 76% (95% CI: 56%–100%) who were confirmed to be negative for the *KIAA1549–BRAF* fusion, although this difference was not statistically significant.

## Discussion

Previous studies have revealed that *KIAA1549–BRAF* fusions are a hallmark mutation in pediatric pilocytic, pilomyxoid, and diffuse astrocytomas of the brain.^20^ Other studies have demonstrated that *BRAF*^*V600E*^ mutations are common in grade 2 pleomorphic xanthoastrocytomas, pleomorphic xanthoastrocytomas with anaplasia, grade 1 gangliogliomas, grade 3 anaplastic gangliogliomas, and pilocytic astrocytomas in the brain. More specifically, *BRAF* rearrangement has been found in 75%–80% of cerebellar pilocytic astrocytomas, and *BRAF*^*V600E*^ mutations have been identified in non-cerebellar regions of the brain.^21^ Most recently, a large analysis of LGG (which includes the spinal LGG analyzed in this study) demonstrated how molecular studies can be used to stratify these tumors into risk categories.^18^ To date, there are few studies specifically addressing the presence of *KIAA1549–BRAF* fusions and *BRAF*^*V600E*^ point mutations in low-grade spinal cord tumors. One study by Gessi et al.^22^ found that spinal gangliogliomas do not harbor the *BRAF*^*V600E*^ mutation in high frequency. Another study by Wang et al.^23^ acknowledged that very little work has been done in regards to understanding the role of BRAF mutations in spinal cord tumors. Ryall et al.^18^ reported the presence of both *BRAFV660E*, *FGFR1* single nucleotide variants, as well as *KIAA1549–BRAF* and *FGFR1–TACC1* fusions in spinal cord LGG. Our data support these previous studies and add to the overall understanding of the genetic landscape of these tumors.

*BRAF* fusions are often correlated with a favorable prognosis in cerebellar LGG. One group, in particular, found that pediatric patients who underwent STR but harbored *KIAA1549–BRAF* fusion had a positive clinical outcome in comparison to patients who had STR and were negative for *KIAA1549–BRAF* fusion.^20^ Our results support these findings. Of the 13 patients with confirmed *KIAA1549–BRAF* fusion, none died during follow-up, regardless of treatment group. Of the 18 patients who confirmed negative for the *KIAA1549–BRAF* fusion, 3 of the patients died putting the 5-year estimated survival rate at 76% with 7 patients experiencing a relapse. This further supports that identification of genetic mutations and fusions in pediatric spinal LGG may be useful in prognostication^18^ and provide information for therapy choices as more targeted therapies are being studied.

There is increasing use of targeted therapy against *BRAF* alterations. A number of clinical trials are evaluating the efficacy of inhibitors of the RAS/RAF/MEK/ERK signaling pathways as well as small molecule inhibitors of *BRAF*^*V600E*^ in these tumor types (NCT01748149).^19^ The specific *BRAF* alteration is correlated to response to treatment; *KIAA1549–BRAF* fusions are RAF-independent and *BRAF*^*V600E*^ mutations are responsive to autophagy and small molecule inhibitors.^11,12,14^ Two of the tumors evaluated in this study were found to harbor *BRAF*^*V600E*^ and would have the potential for BRAF-targeted inhibition. Identification of *KIAA1549–BRAF* fusion mutations could help to inform treatment decisions to include MEK inhibition. Recent data have demonstrated significant responses to MEK inhibition in this patient population.^24^

The primary aim of this study was to investigate the prevalence of *BRAF* mutations and fusions in low-grade spinal cord tumors. Additional mutations were found in *FGFR1*, *CDKN2A*, *H3F3A*, *TP53*, and *PTPN11*. This is consistent with previous reports of alternate mutations found in LGG, but in far less frequency.^3–6,25^ Research has found that some low-grade intracranial tumors harbor alterations in *FGFRs* which involve fusions with TACC genes and FGFR1 tyrosine kinase domain duplication (FGFR1-TKDD) resulting in upregulation of MAPK/ERK and the PI3K pathway.^6,18,26^ Similarly, our data found a *FGFR1* mutation in a patient diagnosed with grade 2 astrocytoma NOS who experienced a relapse of this primary tumor 3 months from diagnosis but has survived to 60 months since diagnosis. Another patient with a pilocytic astrocytoma had a confirmed FGFR1 mutation. Genetic screening studies have found that FGFR1-TKDDs are found predominantly in diffuse gliomas,^6,27^ although it can also be seen in other tumors such as pilocytic astrocytomas^26^ and dysembryoplastic neuroepithelial tumors.^28,29^ Recently, the Consortium to Inform Molecular and Practical Approaches to CNS tumor Taxonomy—Not Official WHO (cIMPACT-NOW) reported that diffuse gliomas characterized by FGFR1 alterations occur primarily in children and that these should be classified as diffuse glioma, FGFR1-mutant tumors.^30^ A comprehensive evaluation of LGG of the CNS found FGFR1 mutations in 1.5% of tumors analyzed and that these often co-occurred with other genetic alterations.^18^ FGFR1-activating mutations and fusions have also been reported in pediatric spinal tumors.^18,31^ At present, there are *FGFR* inhibitors in a clinical trial including erdafitinib which is being studied in the current pediatric MATCH Trial (NCT03210714).

In a previous study, 1.9% of patients with low-grade spinal cord tumors demonstrated mutations in *H3F3A*.^6^  *H3F3A K27M* has proven to be a hallmark of many HG midline gliomas and occurs in about 20% of pediatric glioblastomas.^32^ It was detected in one of our patients diagnosed with a WHO grade 1 ganglioglioma. This patient experienced a relapse and was subsequently diagnosed with an anaplastic ganglioglioma WHO grade 3 and ultimately passed away 19 months from the original diagnosis. This would suggest a concern for a poor clinical outcome with this mutation regardless of primary pathologic diagnosis. At present, there are no treatments to specifically target this mutation but could have potential importance in the prognosis of these tumors.^33^

Limitations of this study include the small patient cohort. Another limitation was that as a retrospective study, some samples were old and that was coupled with suboptimal fixation methods resulting in poor quality DNA and RNA for mutational and fusion assays.^34^ A last potential limitation to this study is that diagnoses were made strictly based off of histology rather than based on other molecular profile data.

The genetic aberrations reported here add to the available information to better understand the molecular underpinnings of low-grade spinal cord tumors. Knowledge about the distinct genetic landscape of these tumors could potentially help to inform treatment choices and predict overall prognosis in patients with pediatric spinal tumors.

## Supplementary Material

vdaa103_suppl_Supplementary_Figure_1

## Notes

**Authorship Statement.** Conception/design of the work: S.T.G. and J.M.M.L. Data collection: S.T.G., L.N., K.D.D., D.L.A., L.H., S.Z., A.M., A.N., M.C., A.L.G., N.F., R.V., T.C.H., M.H.H., S.R., C.H., B.K.D., U.T., and J.M.M.L. Data analyses/interpretation: S.T.G., L.N., K.D.D., K.R.C., D.L.A., C.H., and J.M.M.L. Drafting of the article: S.T.G., K.R.C., and J.M.M.L. Critical revision of the article: S.T.G., K.R.C., and J.M.M.L. Final approval of the article: All authors.
