# Supplementary material for: Clinical and molecular characterization of a multi-institutional cohort of pediatric spinal cord low-grade gliomas
Source: Neurooncol Adv. 2020 Aug 24;2(1):vdaa103. doi: 10.1093/noajnl/vdaa103 (PMC7542983; doi:10.1093/noajnl/vdaa103)
Supplement: vdaa103_suppl_Supplementary_Figure_1 [file vdaa103_suppl_supplementary_figure_1.pdf]

Supplemental 1: 67 gene panel that was used for genetic testing.

ABL1	ERBB3	IDH2	PIK3CA
AKT1	ERBB4	JAK2	PIK3R1
ALK	ESR1	JAK3	PTEN
APC	EZH2	KDR	PTPN11
ATM	FBXW7	KIT	RB1
AURKA	FGFR1	KRAS	RET
BRAF	FGFR2	MAP2K1	RHOA
CCND1	FGFR3	MDM2	ROS1
CCNE1	FLT3	MET	SMAD4
CDH1	FOXL2	MLH1	SMARCB1
CDK4	GNA11	MPL	SMO
CDKN2A	GNAQ	MYC	SRC
CSF1R	GNAS	MYCN	STK11
CTNNB1	H3F3A	NOTCH1	TERT
DDR2	HNF1A	NPM1	TP53
EGFR	HRAS	NRAS	VHL
ERBB2	IDH1	PDGFRA	
